# Possible Mechanisms by Which Topical 5-Fluorouracil and Dermabrasion Could Induce Pigment Spread in Vitiligo Skin: An Experimental Study

**DOI:** 10.1155/2013/852497

**Published:** 2013-04-09

**Authors:** Y. Gauthier, T. Anbar, S. Lepreux, M. Cario-André, L. Benzekri

**Affiliations:** ^1^Department of Dermatology, Saint-André Hospital, 33000 Bordeaux, France; ^2^Department of Dermatology, Al-Minya University, Al-Minya 61111, Egypt; ^3^Department of Pathology, Pellegrin Hospital, 33000 Bordeaux, France; ^4^National Reference for Rare Skin Diseases, University Hospital, 33000 Bordeaux, France; ^5^Department of Dermatology, Mohammed V Souissi University, Ibn Sina Hospital, 8007 Rabat, Morocco

## Abstract

The combination of skin ablation and 5-Fluorouracil (5-FU) ointment was previously tried in the treatment of vitiligo, and good results were specifically reported in glabrous skin without follicular melanocyte reservoirs. *Methods*. This study was carried out on the skins of seven guinea pigs: three were treated with mechanical dermabrasion plus topical 5-FU in an achromic area contiguous to a pigmented area; two were treated by only dermabrasion in a similar area; and two were treated by topical 5-FU alone. Clinical, histological, and ultrastructural studies were performed over two months. *Results*. In guinea pigs treated with dermabrasion plus 5-FU, we observed firstly a delay of wound healing with an obvious inflammatory reaction, and, after two months, evident pigment spread from the pigmented into the achromic area. After six months, we noticed black hair regrowing in the achromic area. Pigment spread was not seen in the guinea pigs skin treated by either dermabrasion or topical 5-FU. We suggest that the inflammatory mediators and enzymes (metalloproteinases), which are locally released over a long time, could stimulate and facilitate melanocyte proliferation and migration through the enlarged intercellular spaces of the epidermis. This sequence of events may be applied to vitiligo patients treated with 5-FU on ablated lesions.

## 1. Introduction

Vitiligo affects 1% of the world's population and probably has a higher incidence in dark-skinned individuals. Three repigmentation patterns are usually described: perifollicular, when the predominant repigmentation is follicular; marginal, when the predominant repigmentation is from the borders of patches; and diffuse, when repigmentation occurs across the patches of vitiligo [1]. Nb-UVB phototherapy is considered to be a very important modality in vitiligo treatment. Its efficacy is mainly due to the stimulation of the perifollicular repigmentation and rarely to the repigmentation from the edges, so treatment of glabrous areas localized on the elbows, the dorsa of the hands, fingers, wrists, and feet is presently challenging. 

Application of 5-Fluorouracil (5-FU) after mechanical dermabrasion, as a treatment for vitiligo, was introduced by Tsuji and Hamada in 1983 [2], and its efficacy was successively confirmed by other authors [3–5].

Successful repigmentation of areas devoid of hair follicles (periungueal areas and the dorsa of the feet) was first reported by Anbar after ER: YAG laser skin ablation followed by topical application of 5-FU and Nb-UVB phototherapy [6–8]. At first sight, this effective association may seem surprising and paradoxical. The mechanism of the action of this combined treatment of vitiligo patients may be better understood by briefly summarizing the previous pharmacological, clinical, and experimental studies related to 5-FU. 

Pharmacologically, 5-FU is an antimetabolite analogue of the naturally occurring pyrimidine uracil which is metabolised via the same metabolic pathways as uracil [9]. Due to its antimitotic activity, it is easy to understand that topical 5-FU is a useful therapy for the treatment of many dermatological disorders characterized by a high mitotic rate (e.g., nonmelanoma skin cancers, actinic keratosis, benign tumours, nail psoriasis, and porokeratosis) [10–12]. In the same way, it is expected that 5-FU applied to a wound will strongly inhibit wound epithelialization and, consequently, will delay the wound healing [13], so it is surprising that 5-Fu would be implicated in the vitiligo repigmentation process which obviously needs melanocyte proliferation.

Clinically, localized hyperpigmentations have been reported during systemic treatment of various cancers by 5-FU. Usually, these hyperpigmented lesions are located on the normally pigmented extremities (hands and feet) and tongue. It has been postulated that these hyperpigmentations could be considered as postinflammatory hyperpigmentations on sites submitted to repeated friction [14–20]. 

The most interesting information about the biological impact of 5-FU on melanocytes has been obtained by experimental studies. In the presence of low concentrations of 5-FU, keratinocytes are selectively destroyed within three weeks, while melanocytes continue to multiply and to form pigment [21]. This different effect of 5-FU on the multiplication of keratinocytes and melanocytes was proposed by Tsuji and Karasek for obtaining cultures of melanocytes free from other nondendritic epidermal cells [22]. From these two experimental studies, it could be concluded that in vitro and probably in vivo nontumoural melanocytes are much less vulnerable to 5-FU than keratinocytes are.

Following this short review, we do not know the exact role of 5-FU in the improvement of the vitiligo repigmentation following dermabrasion. The aim of our study was to explore the possible role of dermabrasion, 5-FU, and their combination on pigment spread on guinea pig skin.

## 2. Material and Methods

### 2.1. Animal Model

We chose guinea pigs since the epidermis of these animals is very similar to that of a man. In guinea pig skin, melanin is transferred from melanocyte processes to surrounding keratinocytes as in human skin [23]. This study involved seven male guinea pigs with patchy black and white fur. In this “piebald-like pattern,” the areas of black skin with black hairs are clearly distinct from the areas of white skin with white hairs. The hairs of the flanks were shaven in areas including pigmented skin contiguous to white achromic skin to assess the pigment spread from the pigmented area into the white area. Treatment of the animals followed the national guidelines (Agreement no.: R-45GRETA-F1-04).

### 2.2. Drug Treatment and Dermabrasion

Approximately, 20 cg of commercially available 5% 5-Fu cream (Efudix Medapharma Paris France) was applied once daily for two days, like in patients, on an area located on the flank overlapping the black and white skin (from 1 mm inside the pigmented area to the entire dermabraded area).

A dermabrader unit (F-319 Silverfox Malea France) with an average speed of 15,000 rpm and diamond fraises of various sizes and shapes were used for the procedure. The superficial dermabrasion was performed under local anaesthesia in at least two different directions and was stopped when uniform punctuate capillary bleeding from the dermal papilla could be seen. Finally, a total surface area of around 6 cm^2^ was dermabraded.

### 2.3. Study Design

The animals were randomly divided into three groups, as detailed in Table 1.

**Group 1**

**Figure 1:**
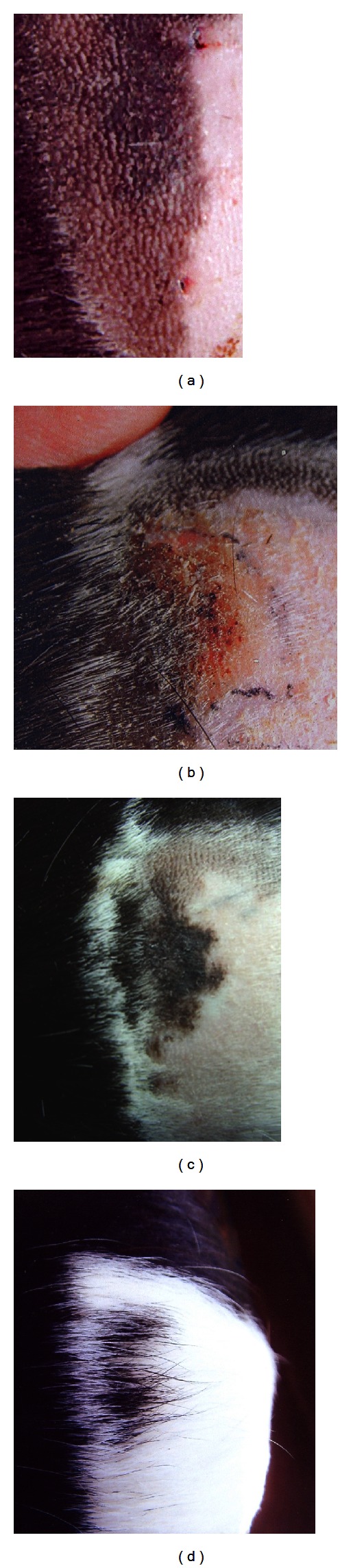
Pigment spread from the pigmented area towards the achromic area. (a) Area including contiguous pigmented and achromic areas before treatment, (b) inflammatory reaction 2 weeks after dermabrasion + topical 5FU cream, (c) pigment spread 4 months after dermabrasion + topical 5FU, and (d) black hairs growth 6 months after treatment.

**Figure 2:**
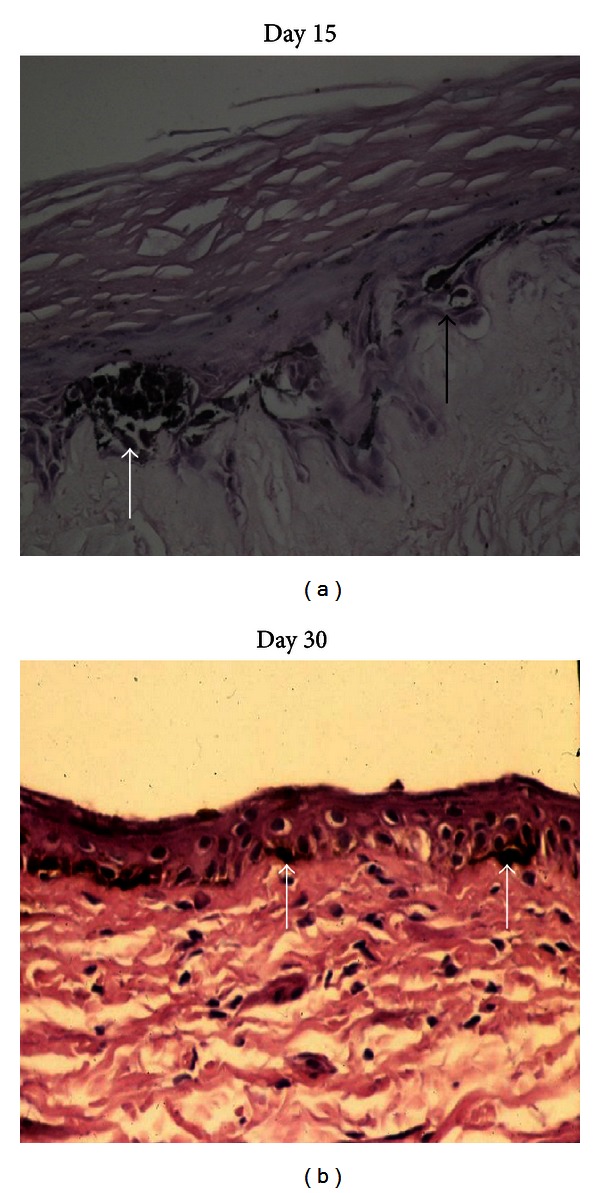
Histological study of pigment spread after dermabrasion + 5-FU ointment. (a) *Day 15*. Disrupted aspect of the epidermis, enlarged intercellular spaces, clustered melanocytes at the margin, (white arrow), and migrating melanocyte with a long dendrite from the margin (black arrow) (Dopa 400x). (b) *Day 30*. Thin epidermis with migrating melanocytes from the margin (white arrows) (HES + Dopa 100x).

Three guinea pigs were subjected to mechanical dermabrasion on areas of the flank including white skin (Figure 1(a)). Superficial dermabrasion was performed under local anaesthesia on a shaved area (Figure 2(b)). Topical 5-FU was then applied on the treated area for two days and was covered by sterilized gauze dressing. Topical antibiotic cream was then applied and covered by gauze, and this continued until complete reepithelialization.

**Group 2**

Two guinea pigs were subjected to dermabrasion alone on similar areas under local anaesthesia without topical 5-FU ointment. Topical antibiotic cream was then applied, covered by gauze, and this continued until complete reepithelialization.

**Group 3**

Two guinea pigs were subjected to topical 5-FU once a day for two days only on the shaved area.

### 2.4. Pigment Spread Assessment

Every week, pictures were taken, and planimetry was used, that is, drawing the lesions on transparent paper. We compared the pigment spread in the followup every month until the end of the study. Followup was done clinically and histologically (with the help of light and electron microscopes).

### 2.5. Pathology

We performed biopsies from the margin including pigmented and achromic skin just after healing of the dermabraded area and at the beginning of pigment spread. Skin samples from the margin were removed under local anaesthesia with 4 mm punches on days 15 and 30 after the dermabrasion.

For light microscopy, tissues were fixed in formal saline and then embedded in paraffin wax. The slides were assessed using an Olympus microscope (Olympus Tokyo). Cryostat sections were stained with H.E.S and Dopa for melanocytes. The sections were examined at magnifications of ×400 and under oil immersion ×1000.

For electron microscope study, tissues were fixed in 1.5% glutaraldehyde in a phosphate buffer and routinely processed for transmission electron microscopy. Ultrathin sections were double contrasted and observed using a Tecnai 12 BioTwin transmission electron microscope (Philips Optique Electronique SAS: Limeil Brévannes, France).

### 2.6. Statistics

Due to the small number of animals investigated in this study, only the mean values of different items (pigment spread distance and healing duration) were taken into account.

## 3. Results

**In Group 1**

After the successive stages of inflammation including erosion and crustation, the reepithelization was completed within 15–17 days (Figures 1(a)-1(b)). After complete healing, the erythema persisted for 28 to 30 days. During this period, the pigment spread was easily observed in the erythematous area, finally covering, after four months, the entire dermabraded area (Figure 1(c)). Surprisingly, 6 months after dermabrasion and topical 5-FU, we saw the growth of black hairs in this area, probably due to reciprocal exchanges between the epidermal and follicular melanocyte reservoirs (Figure (1(d)).

*A Histopathological Study of the Margin Was Performed on Days 15 and 30.*

**(i) 15 Days after Dermabrasion and 5-FU Ointment**

**Figure 3:**
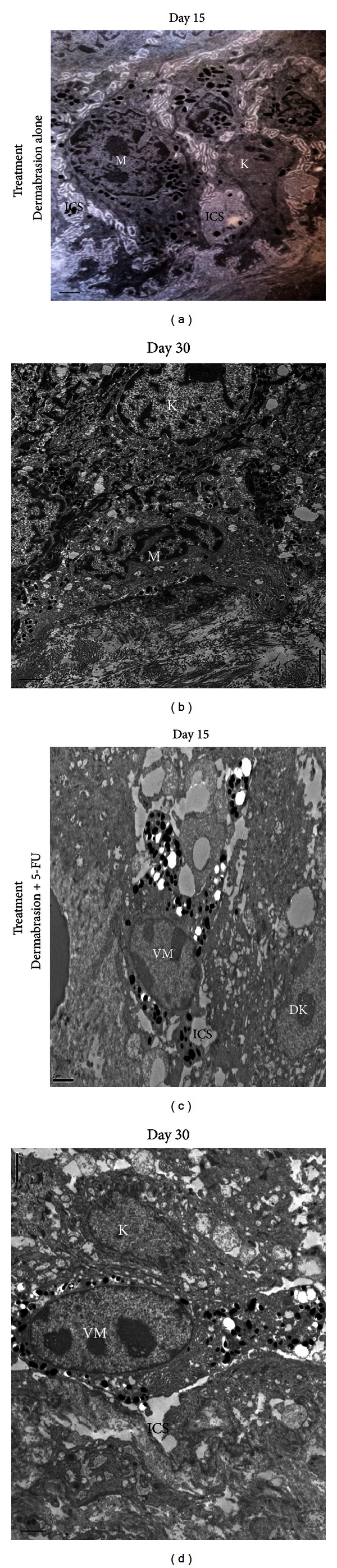
Comparative aspect of melanocytes under electron microscope (linear Scale = 2 *μ*m). *Following dermabrasion alone*: (a) on day 15, after complete healing, normal appearing melanocyte (M) and keratinocytes (K), enlarged intercellular space (ICS); (b) on day 30, normal aspect and cohesion of the epidermis of the margin (×6.000). *Following dermabrasion + 5-Fu:* (c) on day 15, after complete healing, disrupted aspect of the epidermis, damaged keratinocytes (DK) surrounding active melanocyte (VM) with vacuolated cytoplasm, enlarged intercellular spaces (ICS); (d) on day 30, normal appearing keratinocytes (K), migrating melanocyte (VM) with vacuolated cytoplasm, and persistent enlargement of intercellular spaces (ICS) (×6.000).

The epithelialization was complete on day 15 after dermabrasion and 5-FU ointment. Numerous keratinocytes showed few atypical features (atypical nuclear configuration and degenerative changes). This newly regenerative epidermis contained many clustered melanocytes at the margin (Figure 2(a)) and few melanocytes with long dendrites (Figure 2(b)) migrating from the pigmented to the achromic epidermis. In the superficial dermis, a mononuclear cell infiltrate was present. Under an electron microscope, we observed in the treated epidermis many chemically damaged keratinocytes surrounding few active melanocytes, with a vacuolated cytoplasm filled with many melanosomes. Melanocytes were separated from damaged keratinocytes by enlarged intercellular spaces (Figure 3(c)).

**(ii) 30 Days after Dermabrasion and 5-FU Ointment**

We objectivated at the margin that the keratinocytes had a normal appearance. The density of melanocytes in the pigmented epidermis returned to normality. Some bipolar melanocytes were seen crawling towards the achromic epidermis (Figure 2(b)). In the superficial dermis, inflammatory infiltrate was still present. Under an electron microscope, we noted a persistent enlargement of the epidermal intercellular between normal-appearing keratinocytes. Some melanocytes with vacuolated cytoplasm filled with melanosomes were seen migrating between the basal layer and the basement membrane (Figure 3(d)).

**In Group 2**

Following isolated dermabrasion, the wound healing was more rapid, the reepithelialization was obtained within seven days, and the erythema disappeared within 15 days. After four months, the pigment spread from the border separating pigmented and white areas was very discrete, not exceeding 2-3 mms.

*Histopathological Studies of the Margin.*

**(i) 15 Days after Dermabrasion Alone**

The aspect of the epidermis was normal with some persistent enlargement of intercellular spaces. Several normal-appearing melanocytes were observed at the margin of the pigmented area (Figure 2(b)). In the dermis, inflammatory infiltrate was very discrete. Under an electron microscope, we found enlarged intercellular spaces between normal-appearing keratinocytes and active melanocytes without vacuoles in their cytoplasm (Figure 3(a)).

**(ii) 30 Days after Dermabrasion Alone**

The histological aspect of the epidermis was normal, with a reduced width of intercellular spaces between the keratinocytes themselves and between the melanocytes and the basal membrane. Under an electron microscope on day 30, the aspect and the cohesion of the epidermal cells were quite normal (Figure 3(b)).

**In Group 3**

Following only topical treatment with 5-FU for 2 days, we did not observe any clinical or histopathological changes at the treated site, as previously reported [10]. Moreover, no pigment spread from the border was seen.

## 4. Discussion

This study, performed on guinea pig skin, could contribute to better understanding of the possible role of dermabrasion, 5-FU, and their combination in the unexpected improvement of vitiligo repigmentation. This quasisimilarity between the vitiligo lesions and the achromic patches of these piebald-like guinea pigs encouraged us to compare our experimental data to clinical data previously reported in vitiligo patients treated by skin ablation and 5-FU. Before discussing these data, a short review of the repigmentation steps following dermabrasion of normal skin is needed.

### 4.1. Repigmentation Steps after Wounding or Dermabrasion of Normally Pigmented Skin

 In man skin, the epidermal melanin unit includes one melanocyte to approximately 36 keratinocytes. In this functional unit, keratinocytes have an evident regulatory role in skin pigmentation, and the melanocytes may exert an influence on keratinocyte proliferation. However, in guinea pig skin, during the regeneration of the epidermis following injury, melanocytes and keratinocytes seem to react independently [24]. If the basement membrane is not too damaged, keratinocyte migration from the edges or from dermal appendages (hair follicles) can begin as early as a few hours after wounding. Epithelial cells are replaced within three days by division and the mainly upward migration of cells in the stratum basale [25]. A slow repopulation of guinea pig skin by melanocytes mainly issuing from the hair follicle walls is usually seen after complete epithelialization of the wound performed on pigmented skin. The pigment spread from the pigmented area was insignificant, not exceeding 2 mms in all cases.

In human pigmented skin, this difference between keratinocyte and melanocyte behaviour seems greater, and late repigmentation appears more slowly after healing of a skin wound. This may explain the delay in repigmentation that often accompanies wound reepithelialization. The repigmentation of human epidermis following dermabrasion has its main origin in the amelanotic portion of the hair follicle [26]. The process of reepithelization is completed by the eight day. From the tenth day, at the histological level, a few melanocytes begin a well-known centrifugal migration from the infundibulum into the depigmented epidermis [27]. Frequently, the beginning of this repigmentation is observed by the thirtieth day, and total repigmentation may take as long one year [28].

### 4.2. Effect of Isolated Dermabrasion on Pigment Spread from the Margin towards Achromic Area (Group 2)

In guinea pig skin, we did not see any significant pigment spread following only dermabrasion of the achromic area contiguous to pigmented skin. The healing was rapidly obtained within six to seven days without any inflammatory reaction. The pigment spread from the pigmented area was insignificant and did not exceed 2 mms. On day 15, after complete epithelialization, melanocytes could not be found in light and electron microscope observation of the previously dermabraded achromic area. Our data are in a complete agreement with those previously published [2, 7]. 

In vitiligo skin, perifollicular repigmentation but not marginal repigmentation was occasionally reported after exclusively removing the epidermis of macules of vitiligo that retained pigmented hairs [29, 30]. Finally, in guinea pig and human skin, only perifollicular repigmentation can be observed, though rarely, following isolated dermabrasion [31]. 

### 4.3. Effect of Isolated Topical 5-FU Ointment on Pigment Spread from the Margin (Group 3)

In guinea pig skin, we did not observe any local pigment spread following the application of 5-FU cream for two days at the margin separating the pigmented and achromic areas. In the same way in human skin, normal and intact skin did not show any clinical alteration following application of 5-FU cream during topical treatment of many cutaneous disorders (actinic keratosis, psoriasis, and epithelial neoplasms) [10]. Localized hyperpigmentations located in the normally pigmented extremities (hands and feet) and tongue have been reported mainly during systemic treatment of various cancers by 5-FU [14–20] but only occasionally during topical treatment [29, 30]. In vitiligo skin with black or white hairs, no improvement or any pigment spread was observed after the exclusive topical use of 5-FU cream [2].

### 4.4. Effect of Combined Dermabrasion + 5-FU Ointment on Pigment Spread from the Margin (Group 1)

In guinea pig skin, we observed a consequent pigment spread from the edges. Pigment spread began one or two weeks after healing and occurred after four months in all the abraded areas. A growth of black hairs in two animals was noted after six months. Just after the healing, we observed the histological features previously described in a study devoted to inhibition of wound healing by topical 5-FU [13].

In vitiligo skin, the development of pigment spread was quite similar to that we observed in our animal model. In both situations, after dermabrasion or laser ablation followed by topical application of 5-FU for two days, an inflammatory reaction with erythema, erosion, and crustation was noted. Unfortunately in the vitiligo patients investigated, no serial histological studies were performed during or after the treatment. Epithelialization was completed within ten days of the treatment being stopped. Within one to three weeks after the healing, repigmentation began either from the hair follicle and spread centrifugally and either from the margins and spread centripetally. In vitiligo patients, complete or almost complete repigmentation of all treated spots was reported in 64% of cases and partial repigmentation in 18% of cases. The treatment failed in 18% of cases [7]. These failures, which seemed clearly linked to the activity of the vitiligo disease, were not found in the present study because black and white guinea pigs are not vitiligo models. Nb-UVB irradiation was proposed by some authors following the above-mentioned technique to accelerate the pigmentation, but we did not use it in our experimental study. Interestingly, exclusive marginal repigmentation from the edges of glabrous areas was reported in vitiligo patients treated by epidermis ablation and 5-FU ointment [6]. 

### 4.5. Mechanisms by Which 5-FU Could Induce Pigment Spread in Guinea Pig and Vitiligo Skin

Until now, it was difficult to understand why a topical drug, such as 5-FU, well known for its antimitotic activity, could improve the proliferation and migration of melanocytes. Successively, a direct overstimulation of melanocyte proliferation (unproven in melanocytes cultures), an inhibition of agents or cells able to destroy pigment cells, and finally an immunomodulation stabilizing the vitiligo disease [2, 6, 7] (not suitable in guinea pig skin) were evocated.

Based on our clinical and especially our histological findings obtained from this experimental study, we can propose a possible “scenario” including several successive events, allowing us to give an explanation for this enigmatic process. As was previously demonstrated in vitro, 5-FU can exert a selective and differential cytotoxicity according to the type of epidermal cells. Melanocytes seem to be much less vulnerable than keratinocytes to 5-FU, and their ability to proliferate in cultures appears to be preserved [21, 22]. After dermabrasion followed immediately by application of 5-FU ointment for two days, many keratinocytes implicated in the new epithelialization are chemically damaged. Consequently, a strong inflammatory reaction is seen, and the healing is considerably delayed, as has been previously reported [13]. On day 15, after a slow, complete healing, the global aspect of the regenerative epidermis remains disrupted. Some degenerative keratinocytes located in the lower layers still persist. Due to the persistence of a local oedema, the intercellular spaces of the basal layer remain very enlarged for a long time. Active melanocytes with frequently vacuolated cytoplasm are seen migrating through these enlarged intercellular spaces from the pigmented to the achromic epidermis. During this period, inflammatory mediators such as leukotrienes C4 and D4 (LTC4 and LTD4) are locally released, which could stimulate melanocyte proliferation and migration [31–33]. Moreover, during epidermis remodelling, keratinocytes synthesize and secrete metalloproteinases, which are enzymes involved in the degradation of the extracellular matrix. Metalloproteinase 2 has been demonstrated to create a favourable milieu for melanocyte migration [34, 35].These favourable conditions, which persist for a long time, could explain the successful migration of melanocytes from the pigmented to the adjacent achromic area both in guinea pig and vitiligo skin. Possibly, the same sequence of events could be implicated later in the growth of black hairs.

## 5. Conclusion

Neither the topical application of 5-FU nor dermabrasion alone seems to be able to induce any pigment spread either in vitiligo patients or in our animal model. In guinea pig and vitiligo skin, the application of 5-Fu on the dermabraded or ablated epidermis could cause a long-lasting, favourable microenvironment for the melanocyte migration and pigment spread.

## Figures and Tables

**Table 1:** Consequences of different treatments on epithelialization and inflammatory.

Treatment	Time for healing	Inflammatory reaction duration	Pigment spread distance
Topical 5-FU ointment *n* = 2	—	—	0
Dermabrasion alone *n* = 2	7 days	8 days	Few mms
Dermabrasion + 5-FU *n* = 3	15 to 17 days	20 to 27 days	1.5 to 2.5 cms 2 ± 0.5 cm

Reaction and pigment spread. *n*: number of treated guinea pigs.
